# Combined Therapy of Yishen Zhuanggu Decoction and Caltrate D600 Alleviates Postmenopausal Osteoporosis by Targeting FoxO3a and Activating the Wnt/*β*-Catenin Pathway

**DOI:** 10.1155/2022/7732508

**Published:** 2022-07-15

**Authors:** Bole Li, Changqing Jiang, Xinyu Zhan

**Affiliations:** Department of Orthopedics, Zhan's Orthopaedic Hospital of Traditional Chinese Medicine, Hangzhou 310000, China

## Abstract

**Background:**

Postmenopausal osteoporosis (PMO) is the most prevalent metabolic bone disease in women. Yishen Zhuanggu (YSZG) decoction and Caltrate D600 reportedly affects bone formation. This study aimed to investigate the efficacy and mechanism of YSZG decoction combined with Caltrate D600 in PMO treatment.

**Methods:**

Ovariectomy-induced PMO rat model was treated with YSZG or/and Caltrate D600 for 12 weeks. Femur bone mineral density (BMD), osteoporosis-related protein expression, and serum parameters were measured. Pathological features of femur bone tissues were observed using hematoxylin and eosin staining. Serum levels of oxidative stress parameters were measured using corresponding commercial kits. The mRNA and protein expression of FoxO3a, Wnt, and *β*-catenin was detected using qRT-PCR and western blotting.

**Results:**

The BMD and ultimate load of PMO rats were increased after treatment with YSZG. YSZG treatment promoted the bone trabeculae formation of PMO rats. YSZG treatment also induced bone differentiation and suppress oxidative stress in PMO rats, evidenced by the increased BALP, Runx2, OPG, SOD, and CAT levels, as well as the decreased TRACP 5b, RANKL, ROS, and MDA levels. Additionally, YSZG treatment downregulated the FoxO3a expression and upregulated the levels of Wnt and *β*-catenin in PMO rats. Caltrate D600 addition showed an auxiliary effect for YSZG.

**Conclusion:**

YSZG decoction exerts the antiosteoporotic effect on PMO by restraining the FoxO3a expression and activating the Wnt/*β*-catenin pathway, which has an impressive synergistic effect with Caltrate D600.

## 1. Introduction

Postmenopausal osteoporosis (PMO) is a metabolic bone disorder effecting women aged more than 50 years. Osteoporosis is characterized by a low bone mass and a deteriorating microarchitecture of bone tissues [1]. In Europe, approximately 21% women aged 50–84 years are diagnosed as osteoporosis [2], and the lifetime risk of women with PMO is close to 50% [3]. Treatment of osteoporosis is generally recommended for postmenopausal women. At present, bisphosphonates [4], estrogen [5], calcitonin [6, 7], strontium salts [8], and parathyroid hormone [9] are the effective drugs for PMO treatment in clinical practice. However, these drugs may be not suitable for long-term clinical therapy, due to produced adverse effects, including renal injury, endocrine disorders, and intestinal problems [10–12]. Therefore, there is an urgent need to develop a more economical, safe, and comprehensive drugs for PMO treatment.

Traditional Chinese medicine (TCM) has achieved unique and impressive advantages in treating PMO in China [13–16]. TCM believes that the pathogenesis of PMO is associated with the deficiency in kidney function, accompanied by spleen and stomach weakness, insufficient blood supply to liver, and blood stasis. Following these principles, the main TCM treatment for PMO is kidney tonifying [17, 18], which has achieved satisfactory therapeutic effects on kidney function deficiency and osteoporosis [18]. TCM achieves higher efficacy in increasing the spine bone mineral density (BMD) compared with western medicines in women with PMO [15]. Other studies have highlighted the advantages of TCM in improving the BMD of patients with primary osteoporosis [19].

TCM can regulate the body's internal environment in a multistep and multitarget manner [20, 21]. Yishen Zhuanggu (YSZG) decoction as a promising TCM formula can relax muscles and tendons. Caltrate D600 is a western medicine used for osteoporosis treatment. The therapeutic mechanism of YSZG decoction combined with Caltrate D600 in improving PMO remains unknown. In this study, we aimed to investigate the efficacy and mechanism of YSZG decoction combined with Caltrate D600 in the treatment of PMO.

## 2. Materials and Methods

### 2.1. Preparation of YSZG Decoction

YSZG decoction consists of 15 Chinese herbal medicines, including *Cornu Cervi Degelatinatum*, *Cuscuta chinensis*, *Cyathula officinalis kuan*, *Angelica sinensis*, *Szechuan Lovage Rhizome*, fried *Eucommia ulmoides*, *Rhizoma Drynariae*, *Astragalus membranaceus*, *Caulis spatholobi*, *Taraxacum mongolicum*, *Rhizoma cibotii*, *Cortex Moutan*, *Ligustrum lucidum* Ait, *Eclipta alba*, and dried *tangerine* peel. These herbal medicines were immersed in water (1 : 2 v/v) and boiled to the concentration of 1 g/mL. The final decoction was stored at 4°C prior to treatment.

### 2.2. Animals and Treatment

Forty female Sprague-Dawley rats (240 ± 10 g, 6-month-old; Shanghai SLAC Laboratory Animal Co., Ltd., China) were maintained under a standard condition (3-4 rats per cage; 50%–65% humidity at 22–25°C with a natural day/night cycle). All animals were fed with basal 5L79 diets (PMI nutrition, MO, USA) and free access to distilled water during experiment. Ovariectomized surgery was performed on 30 female rats randomly selected from a group of 40 rats. In brief, rats were anesthetized by intraperitoneally injecting with 30 mg/kg pentobarbital sodium, and then bilateral ovaries were removed, followed by suturing wound. Four weeks later, rats underwent ovariectomy were assigned to three groups: the model group, the YSZG group, and the YSZG + Caltrate D600 group. Sham ovariectomy was performed in the sham group (*n* = 10) by removing adipose tissues surrounding the ovary. Rats in the sham and model groups were treated with normal saline at an oral dose of 10 mL/kg/d for 12 weeks. Rats in the YSZG group were intragastrically administered with YSZG decoction (1 mL/100 g body weight) for 12 weeks. Rats in the YSZG + Caltrate D600 group received YSZG decoction (1 mL/100 g body weight) and Caltrate D600 tablet (5 mg/100 g) by intragastrical administration for 12 weeks. Animal experiments were approved by the IRB of Zhan's Orthopaedic Hospital of Traditional Chinese Medicine.

### 2.3. BMD Measurement

After treatment for 12 weeks, rats were anesthetized by intraperitoneally injecting with 30 mg/kg pentobarbital sodium. The BMD of femurs was detected using dual-energy X-ray absorptiometry (Medix DR, France).

### 2.4. Three-Point Bending Test

Mechanical properties of the femoral diaphysis were evaluated by a three-point bending test. One hour later, left femurs of rats were placed under a vertical load. The load was increased at a rate of 2 mm/min until the bone broke, and the ultimate load was recorded.

### 2.5. Histological Analysis

Femur bone tissues were fixed, decalcified, and embedded in paraffin. Prior to the histopathological analysis, tissue slides (5 *μ*m) were dewaxed and incubated with hematoxylin and eosin (H&E) staining (Beyotime, China). A BX53 light microscope (Olympus, Japan) was used for imaging.

### 2.6. Measurement of Serum Parameters

Blood samples were collected from rats after 8 h of fasting. Serum levels of estradiol (*E*_2_), bone-specific alkaline phosphatase (BALP), runt-related transcription factor 2 (Runx2), tartrate-resistant acid phosphatase 5b (TRACP 5b), malondialdehyde (MDA), superoxide dismutase (SOD), and catalase (CAT) were measured using corresponding enzyme-linked immunosorbent assay (ELISA) kits (Shanghai Enzyme-Linked immunization, China). ROS level in serum was determined using the Cellular ROS Assay Kit (Abcam, UK).

### 2.7. qRT-PCR

Total RNA was extracted from bone tissues using the EASYspin Plus Bone Tissue RNA Kit (Aidlab, China) following the manufacturer's instructions. HiScript reverse transcriptase (Vazyme, China) was used for reverse transcription of RNA into first-strand cDNA. qRT-PCR was carried out using the SYBR Green Master Mix (Vazyme) under the ABI 7500 fast real-time PCR (Applied Biosystems, Foster City, CA, USA). PCR amplification was conducted as follows: 95°C for 4 min; 38 cycles of 95°C for 20 s, 60°C for 30 s, and 72°C for 30 s. Relative mRNA expression was calculated using the 2^−△△Ct^ method. GAPDH was used as a reference gene for the PCR analysis. Primer sequences are listed Table S1.

### 2.8. Western Blotting

Total protein was extracted from bone tissues using the bone tissue protein extraction kit (BestBio, China) following the manufacturer's instructions. Concentrations of proteins were measured using a BCA protein assay kit (Thermo Fisher Scientific, IL, USA). For western blot analysis, proteins were separated using SDS-PAGE and transferred to polyvinylidene difluoride (PVDF) membranes (Millipore, MA, USA). Subsequently, PVDF membranes carrying target peptides were blocked in 5% skim milk (Beyotime, China) and then incubated with primary antibodies against RANKL (1 : 1,000; PA5-110268, Invitrogen, CA, USA), OPG (1 : 1,000; ab73400, Abcam, UK), FoxO3a (1 : 1,000; ab23683, Abcam), Wnt1 (1 : 3,000; PA5-85217, Invitrogen), *β*-catenin (1 : 5,000; ab73400, Abcam), and GAPDH (1 : 10,000; ab181602, Abcam) at 4°C overnight. Next, membranes were incubated with secondary antibody Goat Anti-Rabbit IgG H&L (HRP) (1 : 2,000; ab6721, Abcam) for 2 h. Band intensity of protein peptide was detected using the enhanced chemiluminescence system with the ImagePro Plus software.

### 2.9. Statistical Analysis

All data were presented as mean ± standard deviation. Data analysis was conducted using the GraphPad Prism 5 (GraphPad Software, CA, USA). Differences between two groups were analyzed using Fisher's exact test. Differences between groups were analyzed using unpaired *t*-test, and those across four groups were analyzed using one-way analysis of variance. *P* < 0.05 was statistically significant.

## 3. Results

### 3.1. YSZG Decoction Increases the Levels of *E*_2_ and BMD in PMO Rats

**Figure 1:**
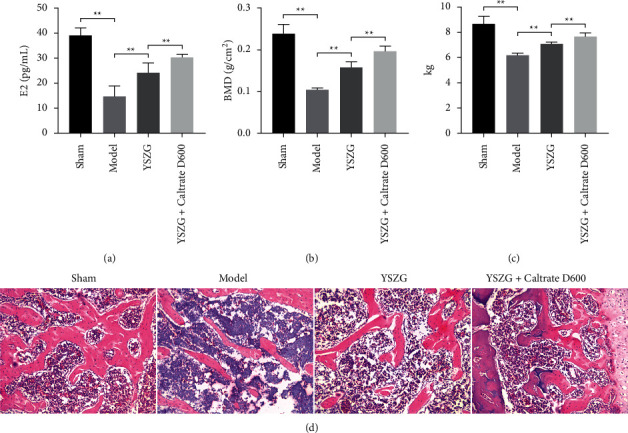
Yishen Zhuanggu (YSZG) decoction promotes the bone microstructure and density of postmenopausal osteoporosis (PMO) rats. (a) Serum level of estradiol (E2) in PMO rats. (b) The bone mineral density (BMD) of rat femur. (c) Ultimate load of rat femurs. (d) Hematoxylin and eosin staining of rat femur tissues. PMO rats were established by ovariectomy and treated with YSZG decoction or/and Caltrate D600. Data were presented as mean ± standard deviation (SD) (*n* = 10). ^*∗∗*^*P* < 0.01.

PMO rat model was established by ovariectomy, and bone-linked markers (E2 and BMD) were determined. Deficiency of postmenopausal *E*_2_ contributes to the decline of BMD and the risk of osteoporosis [22]. ELISA showed that the serum levels of *E*_2_ were significantly decreased in PMO rats compared with that in sham rats (*P* < 0.01). YSZG treatment significantly increased the serum *E*_2_ levels in PMO rats, and Caltrate D600 enhanced the effect of YSZG (*P* < 0.01; Figure 1(a)). Meanwhile, we found that the femur from PMO rats had a lower BMD than that from sham rats, whereas YSZG decoction significantly increased the BMD of femur in PMO rats (*P* < 0.01). Combined treatment of YSZG and Caltrate D600 showed an obvious synergistic effect on improving the femur BMD in PMO rats (*P* < 0.01; Figure 1(b)).

### 3.2. YSZG Decoction Enhances Bone Mechanical Properties and Bone Trabeculae Formation of PMO Rats

To further investigate whether YSZG decoction improves the bone mechanical properties of PMO rats, maximum bone stress was measured. As shown in Figure 1(c), femurs from PMO rats showed a marked decrease in ultimate load compared with that from sham rats (*P* < 0.01). A substantial increase in the ultimate load was observed in PMO rats after administration with YSZG decoction, and YSZG + Caltrate D600 treatment presented better effect (*P* < 0.01; Figure 1(c)). Moreover, H&E histological analysis was performed to observe the morphology of bone trabeculae in PMO rats. Compared with the sham group, the femur tissues from PMO rats showed the obvious damage in the trabecular integrity and microarchitecture and the increased osteoclast number. YSZG decoction treatment significantly restored the trabecular integrity and decreased the number of osteoclasts in femur tissues of PMO rats, and Caltrate D600 addition potentiated the effect of YSZG (Figure 1(d)).

### 3.3. YSZG Decoction Promotes the Bone Formation and Resorption in PMO Rats

**Figure 2:**
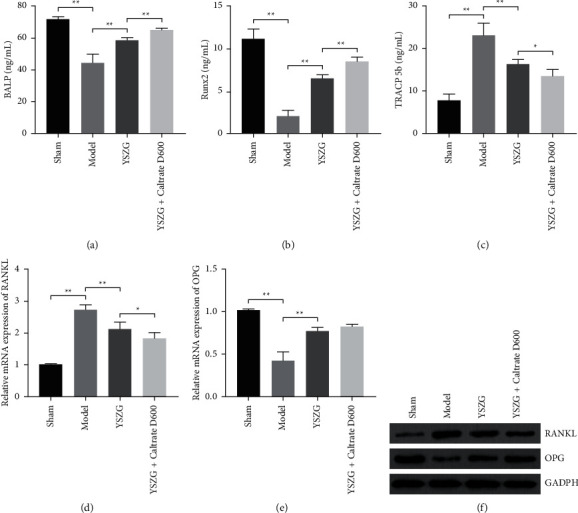
YSZG decoction promotes the bone formation and resorption in PMO rats. (a–c) Serum levels of BALP, Runx2, and TRACP 5b in rats. (d–f) Relative mRNA and protein expression level of RANKL and OPG in bone tissues of rats. PMO rats were established by ovariectomy and treated with YSZG decoction or/and Caltrate D600. Data were presented as mean ± SD (*n* = 10). ^*∗*^*P* < 0.05 and ^*∗∗*^*P* < 0.01.

BALP is an important biomarker for bone formation, and Runx2 is a critical transcription regulator in osteoblast differentiation [23, 24]. As shown in Figures 2(a) and 2(b), the levels of BALP and Runx2 in PMO rats were evidently lower than that in sham rats (*P* < 0.01). YSZG treatment remarkably decreased the levels of BALP and Runx2 in PMO rats, and Caltrate D600 addition potentiated the effect of YSZG (*P* < 0.01; Figures 2(a) and 2(b)). In addition, TRACP 5b is a bone resorption marker, the level of which in PMO rats was markedly higher than that in sham rats, whereas YSZG or/and Caltrate D600 treatment decreased the TRACP 5b level in PMO rats (*P* < 0.05; Figure 2(c)). Moreover, RANKL is an inducer of osteoclastogenesis, and OPG is a decoy receptor of RANKL [25]. qRT-PCR and western blotting presented that the expression of RANKL was upregulated in PMO rats, while OPG was downregulated (*P* < 0.01). YSZG treatment significantly decreased the RANKL level and increased the OPG level in PMO rats, and Caltrate D600 addition enhanced the inhibitory effect of YSZG on RANKL expression (*P* < 0.05; Figures 2(d)–2(f)).

### 3.4. YSZG Decoction Inhibits the Oxidative Stress of PMO Rats

**Figure 3:**
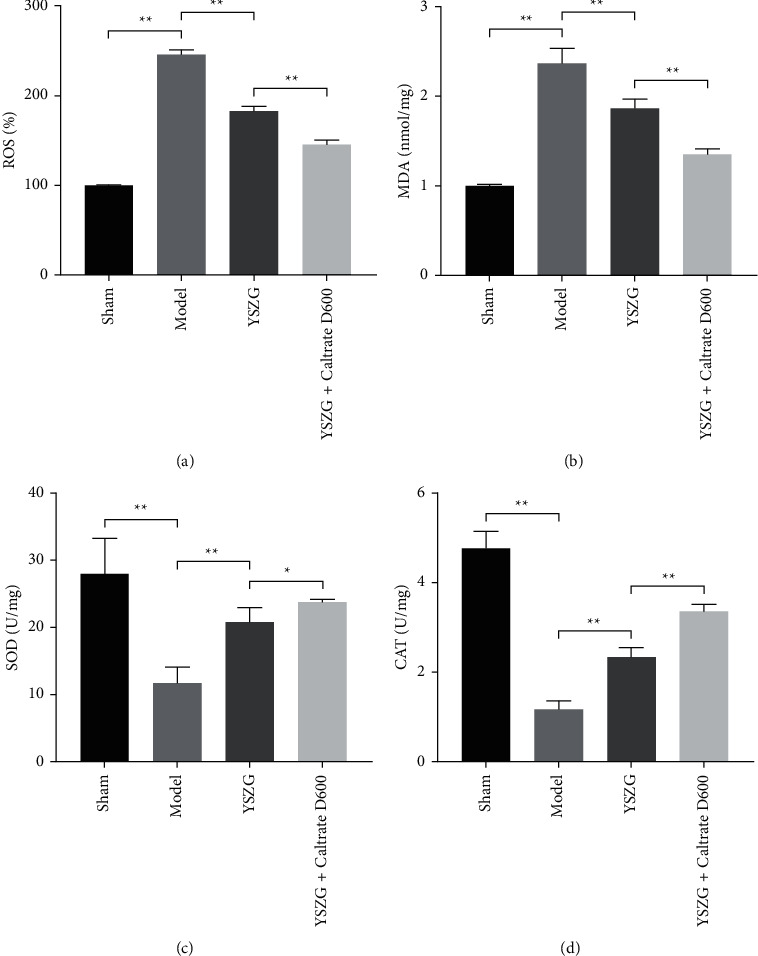
YSZG decoction inhibits the oxidative stress in PMO rats. (a–d) Serum levels of oxidative stress-related biomarkers (ROS, MDA, SOD, and CAT) in rats. PMO rats were established by ovariectomy and treated with YSZG decoction or/and Caltrate D600. Data were presented as mean ± SD (*n* = 10). ^*∗*^*P* < 0.05 and ^*∗∗*^*P* < 0.01.

Oxidative stress is inevitably implicated in the pathological processes of PMO, and ROS, MDA, SOD, and CAT are important biomarkers related to oxidative stress [26]. ELISA showed that the serum levels of ROS and MDA were significantly higher in PMO rats than that in sham rats (*P* < 0.01; Figures 3(a) and 3(b)). Conversely, serum levels of SOD and CAT were decreased in PMO rats (*P* < 0.01; Figures 3(c) and 3(d)). YSZG decoction significantly reduced the ROS, MDA levels and increased the SOD and CAT levels in PMO rats, and Caltrate D600 enhanced the antioxidative effect of YSZG (*P* < 0.05; Figures 3(a)–3(d)).

### 3.5. YSZG Decoction Suppresses the FoxO3a Expression and Activates the Wnt/*β*-Catenin Pathway in PMO Rats

**Figure 4:**
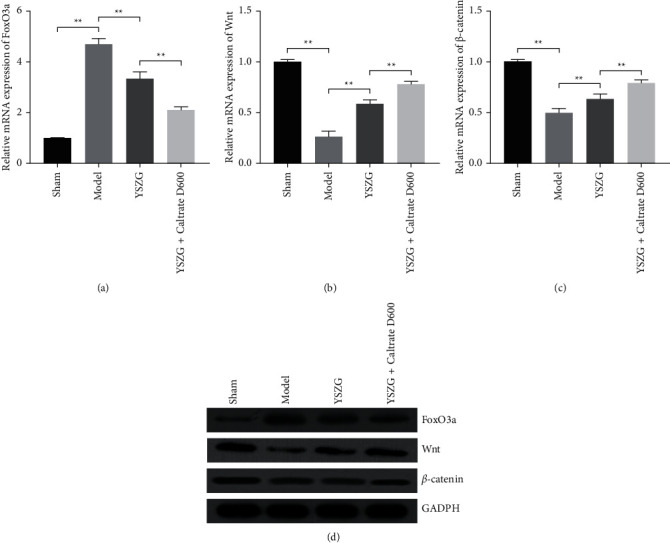
YSZG decoction suppresses the FoxO3a expression and activates the Wnt/*β*-catenin pathway in PMO rats. (a–c) Relative mRNA expression of FoxO3a, Wnt, and *β*-catenin in rats. (d) Relative protein levels of FoxO3a, Wnt, and *β*-catenin. PMO rats were established by ovariectomy and treated with YSZG decoction or/and Caltrate D600. Data were presented as mean ± SD (*n* = 10). ^*∗∗*^*P* < 0.01.

FoxO3a is an essential transcription factor regulating the osteoblastic differentiation in PMO [27]. qRT-PCR and western blotting presented that the expression of FoxO3a was significantly increased in PMO rats compared to that in sham rats (*P* < 0.01). YSZG decoction treatment markedly downregulated the level of FoxO3a in PMO rats, and Caltrate D600 enhanced the effect of YSZG (*P* < 0.01; Figures 4(a)–4(d)). In addition, Wnt/*β*-catenin pathway is also crucial for osteoblastic differentiation and bone loss prevention, which is interfering by FoxO3a [28]. Our results presented that the expression of Wnt and *β*-catenin dramatically decreased in PMO rats when compared to that in sham rats (*P* < 0.01). YSZG administration upregulated the Wnt and *β*-catenin levels in PMO rats, and Caltrate D600 potentiated the effect of YSZG (*P* < 0.01; Figures 4(b)–4(d)).

## 4. Discussion

TCM has unique advantages in treating PMO in a multistep and multitarget manner. Recently, combined therapy of TCM and western medicines has been considered as a promising therapeutic strategy for PMO. YSZG decoction as a TCM is widely used for enhancing bone strength, which exhibits antiosteoporosis effects [29]. Caltrate D600 is a western medicine that is of assistance in preventing and treating osteoporosis. YSZG decoction and Caltrate D600 both have impressive efficacy in tonifying the kidney and improving osteoporosis [30]. Our present study showed the synergistic effect of YSZG decoction and Caltrate D600 in attenuating osteoporosis in ovariectomized rats. Meanwhile, the potential mechanism of YSZG decoction combined with Caltrate D600 therapy was involved in the downregulation of FoxO3a and the activation of Wnt/*β*-catenin pathway.

PMO is characterized by a sharp decline in estrogen levels following menopause. Meanwhile, the accelerated bone turnover rate and the unbalanced bone metabolism process appear in PMO [31, 32]. Therefore, PMO rat model was established by ovariectomy. *E*_2_ is a female hormone that decreased in postmenopausal women [33, 34], and BMD is a critical parameter of PMO [35]. ELISA showed that the serum *E*_2_ and BMD levels decreased in PMO rats. Pathological damage in the trabecular integrity and microarchitecture and increased osteoclast number exhibited in the femur of PMO rats. These results suggest that PMO rat model was successfully constructed. YSZG decoction treatment increased serum *E*_2_ levels and BMD, restored the trabecular integrity, and reduced osteoclasts in PMO rats. Meanwhile, we noticed that YSZG treatment enhanced the formation of bone trabeculae in PMO rats. Caltrate D600 addition potentiated the repressive effect of YSZG on PMO. These results revealed that YSZG decoction combined with Caltrate D600 therapy had a better therapeutic effect on PMO.

Bone differentiation includes old bone resorption and new bone formation, which are, respectively, involved with two types of cells: osteoclasts and osteoblasts [36]. BALP, Runx2, TRACP 5b, RANKL, and OPG are bone differentiation-related proteins [37–39]. In our study, serum levels of BALP, Runx2, and OPG decreased, while those of TRACP 5b and RANKL increased in PMO rats. YSZG decoction potently upregulated the BALP, Runx2, and OPG expression and inhibited the increase of TRACP 5b and RANKL levels in PMO rats. Caltrate D600 addition to YSZG decoction resulted in an auxiliary antiosteoporotic effect in PMO rats. These findings demonstrate that the antiosteoporotic effect of YSZG decoction is associated with bone metabolism.

Oxidative stress, an indirect effect of estrogen deficiency, which is closely related to bone loss [40, 41]. Sridhar et al. found that estrogen deficiency elevates the levels of hydrogen peroxide and lipid peroxide in the femur and impairs the bone antioxidant systems, thereby accelerating bone loss [42]. Therefore, the levels of oxidative stress biomarkers (ROS, MDA, SOD, and CAT) were measured in the serum. Excessive ROS cause oxidative damage to lipids, proteins, and DNA, thereby yielding stable oxidized biomolecule products, including MDA [43]. SOD and CAT are antioxidant enzymes. The increased ROS and MDA and the reduced SOD and CAT levels were found in PMO rats. Following YSZG treatment, ROS and MDA levels were decreased, whereas SOD and CAT levels were enhanced, which indicated that YSZG decoction may regulate bone metabolism by improving oxidative stress.

FoxOs are key factors affecting the redox balance of bone cells, meanwhile mediating oxidative stress osteoporosis [44]. FoxO3a is a member of FoxOs, which stimulates the expression of functional genes that regulate apoptosis and oxidative stress [45]. Wnt/*β*-catenin signaling pathway is a well-known osteoblastic pathway [46, 47]. Inhibition of the Wnt/*β*-catenin pathway results in abnormal bone metabolism, thus inducing osteoporosis [48, 49]. Activated Wnt/*β*-catenin pathway upregulates the expression of osteoblast markers, such as Runx2, calcification, and ALP activity, and promotes osteoblast differentiation [47]. Under oxidative stress, FoxO can be activated to bind to *β*-catenin and translocate into nucleus, thereby limiting the Wnt/*β*-catenin pathway-mediated transcription and then reducing osteoblast proliferation and differentiation and eventually leading to oxidative stress-induced osteoporosis [50, 51]. Our results showed that YSZG decoction significantly inhibited the expression of FoxO3a and increase the expression levels of Wnt and *β*-catenin, and Caltrate D600 addition enhanced this effect. YSZG decoction combined with Caltrate D600 therapy can effectively inhibit FoxO3a expression and activate the Wnt/*β*-catenin pathway in PMO rats.

## 5. Conclusions

In summary, the present study confirmed the therapeutic efficacy of YSZG decoction combined with Caltrate D600 therapy in improving PMO. YSZG decoction can improve the BMD of PMO rats, regulate bone metabolism, and reduce oxidative stress via suppressing FoxO3a and activating Wnt/*β*-catenin pathway. YSZG decoction can be as an oral medicine for the treatment of PMO, and Caltrate D600 addition may achieve the better therapeutic effect. However, this therapeutic strategy is needed to be validated in clinical practice. Besides, the molecular mechanism for PMO treatment involving in FoxO3a and Wnt/*β*-catenin pathway is needed to be further elucidated.

## Data Availability

The data used to support the findings of this study are available from the corresponding author upon request.

## Supplementary Materials

Supplementary Materials Table S1: primer sequences for qRT-PCR.
