# Supplementary material for: Combined Therapy of Yishen Zhuanggu Decoction and Caltrate D600 Alleviates Postmenopausal Osteoporosis by Targeting FoxO3a and Activating the Wnt/β-Catenin Pathway
Source: Evid Based Complement Alternat Med. 2022 Jul 15;2022:7732508. doi: 10.1155/2022/7732508 (PMC9307327; doi:10.1155/2022/7732508)
Supplement: Supplementary Materials — Table S1: primer sequences for qRT-PCR. [file 7732508.f1.docx]

Table S1: Primer sequences for qRT-PCR.

| Name | Sequence (5’-3’) |
| --- | --- |
| RANKL forward | GTCGCCCTGTTCCTCTACTT |
| RANKL reverse | TTTCTCGGCTCTCAGGTGTT |
| OPG forward | AATGTCCTCCTGGCACCTAC |
| OPG reverse | CCTTCCTCGCATTCACACAC |
| FoxO3a forward | GCACCAATTCTAACGCCAGCAC |
| FoxO3a reverse | ATCCAGCAGGTCGTCCATGAGG |
| Wnt1 forward | CAAGATCGTCAACCGAGGCT |
| Wnt1 reverse | TCACACGTGCAGGATTCGAT |
| β-catenin forward | GCGCCATTTTAAGCCTCTCG |
| β-catenin reverse  GAPDH forward  GAPDH reverse | CTGAAGCTGCTCCTCAGACC GGGAGCCAAAAGGGTCAT  GAGTCCTTCCACGATACCAA |
